# The effect of illustrations on patient comprehension of medication instruction labels

**DOI:** 10.1186/1471-2296-6-26

**Published:** 2005-06-16

**Authors:** Stephen W Hwang, Carolyn QN Tram, Nadia Knarr

**Affiliations:** 1 Centre for Research on Inner City Health, St. Michael's Hospital, Toronto, Ontario, Canada; 2 University of Toronto, Ontario, Canada

## Abstract

**Background:**

Labels with special instructions regarding how a prescription medication should be taken or its possible side effects are often applied to pill bottles. The goal of this study was to determine whether the addition of illustrations to these labels affects patient comprehension.

**Methods:**

Study participants (N = 130) were enrolled by approaching patients at three family practice clinics in Toronto, Canada. Participants were asked to interpret two sets of medication instruction labels, the first with text only and the second with the same text accompanied by illustrations. Two investigators coded participants' responses as incorrect, partially correct, or completely correct. Health literacy levels of participants were measured using a validated instrument, the REALM test.

**Results:**

All participants gave a completely correct interpretation for three out of five instruction labels, regardless of whether illustrations were present or not. For the two most complex labels, only 34–55% of interpretations of the text-only version were completely correct. The addition of illustrations was associated with improved performance in 5–7% of subjects and worsened performance in 7–9% of subjects.

**Conclusion:**

The commonly-used illustrations on the medication labels used in this study were of little or no use in improving patients' comprehension of the accompanying written instructions.

## Background

Health literacy is the ability to read, comprehend, and act on health-related materials such as consent forms, prescription drug labels, and medical instructions [1]. Approximately 44 million Americans are functionally illiterate and another 50 million have marginal literacy skills [2]. In a study conducted at two urban public hospitals, about one-third of patients had inadequate or marginal health literacy, and 42% were unable to understand written directions for taking medication on an empty stomach [3].

Pharmaceutical labels must provide critical instructional information to people with different experiences and education levels. Patients are faced with the responsibility of converting declarative information into procedural application, resulting in a substantial risk of errors [4]. Pictorial instructions may be provided in an effort to make the procedural information more readily accessible and its comprehension less dependent on the individual's background or prior knowledge [5-7]. Relatively little research has been conducted on the cognitive processes involved in the interpretation of written and pictorial instructions on medication labels [4,7].

Special instructions regarding how a prescription medication should be taken or its possible side effects are often applied to medication bottles in the form of auxiliary labels. These labels typically include small illustrations that are intended to enhance comprehension. We conducted this study to determine whether the addition of illustrations to prescription medication instruction labels affects patients' comprehension of the accompanying written information.

## Methods

### Setting and subjects

This study was conducted at three family practice clinics affiliated with an urban academic teaching hospital in Toronto, Ontario. These clinics provide primary care to a large patient population living in the central area of the city. From January to September 2001, consecutive patients presenting to the clinic during regular office hours on selected weekdays were approached and asked to participate in a study of comprehension of prescription labels. Days on which patients were enrolled were selected on the basis of availability of a member of the research team. A total of 130 participants were enrolled. Patients were excluded if they were too ill to participate or were unable to communicate in English. Patients who participated in the study gave written informed consent and received a $5 payment. The St. Michael's Hospital Research Ethics Board approved this study.

### Data collection

**Figure 1:**
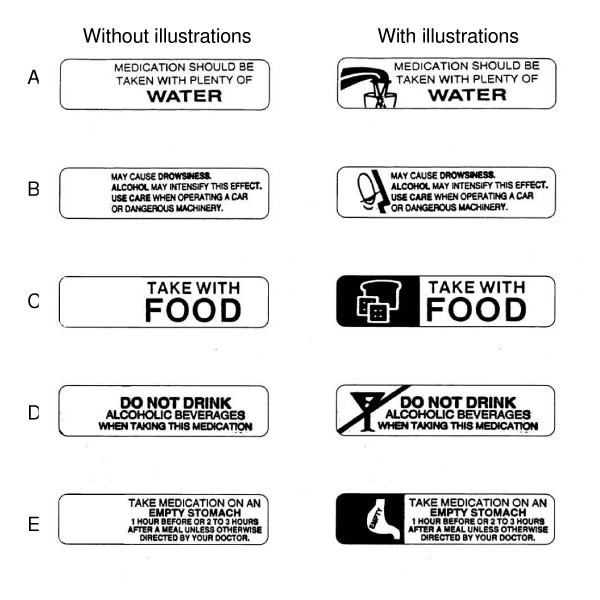
Prescription medication instruction labels.

One of the investigators (CQNT) conducted face-to-face interviews with participants that obtained information on demographic characteristics, native language, and education. Participants were then presented with five instruction labels regarding how certain prescription medications should be taken or their potential side effects (Figure 1). Pharmacies usually affix these labels to the bottles or packaging of certain medications when they are dispensed to the patient. We selected five labels from among those in common use by pharmacies in Toronto. Labels were deliberately chosen to provide a wide range in terms of our assessment of the complexity of both the written information and the accompanying illustration. The labels were presented as black-and-white images that were enlarged from the original label size of 4 × 1 cm to a final size of 8 × 2 cm to enhance readability. The five labels were presented on a single sheet of letter-sized paper.

Participants were first shown five labels with text only (Figure 1, left column), then a separate sheet of paper with the labels with identical text and the addition of visual illustrations (Figure 1, right column). In each case, participants were asked, "If this label were on your pill bottle, how would you take this medication?" Participants were allowed unlimited time to reply. The interviewer wrote down participants' verbatim responses on a survey form; interviews were not audiotaped.

Following presentation of all labels, participants were given the Rapid Estimate of Adult Literacy in Medicine (REALM) test. The REALM test is a previously validated instrument that uses an objective scoring system to assign a grade-range estimate of literacy (grade 0 to 6, grade 7 to 8, or grade 9 and above) [8]. This test is a simple and widely used research instrument that is highly correlated with other measures of health literacy such as the Test of Functional Health Literacy in Adults (TOFHLA) [9].

### Data coding

Two researchers independently coded participants' interpretations of medication labels as incorrect, partially correct, or completely correct. Disagreements in coding were resolved by consensus after further discussion. Labels A, C, and D were deemed to each convey a single main informational component, and interpretations of these labels were coded as either incorrect or completely correct. Labels B and E were deemed to each convey three main informational components. For example, for label B the informational components were: (1) possibility of drowsiness, (2) alcohol may worsen effect, and (3) caution if driving or handling machinery. If none of these components could be identified in the participant's verbatim response, their answer was coded as incorrect. If one or two of these components was found, the response was coded as partially correct. If all three components were found, the response was coded as completely correct. This method of coding, although not previously validated, offered useful detail regarding the completeness of the participants' comprehension. At the time of coding, investigators were blinded to participants' literacy level. The effect of the addition of illustrations on participants' performance was classified as improved, worse, or unchanged, based on the categorization of their first and second responses as incorrect, partially correct, or completely correct. When improvement or worsening was noted, responses were examined to characterize the nature of the change.

### Statistical analyses

The sign test was used to assess whether there was significant improvement or worsening in the interpretation of the label with the addition of the illustration. Chi-square analyses were used to assess whether the effect of the addition of illustrations on participants' performance on label interpretation (improved, worse, or no change) was significantly associated (p < 0.05) with sex, age (under 25 years, 25 to 39 years, 40 to 64 years, or 65 years and over), native language (English or other language), or grade-range estimate of health literacy (assessed using the REALM). All significance tests were two-sided. Statistical analyses were performed using SPSS 10.0 (SPSS, Inc., Chicago, IL).

The power of this study was dependent on both the number of individuals whose performance on label interpretation changed with the addition of illustrations, and the anticipated magnitude of this effect. Assuming that about 25% of subjects or 30 individuals would demonstrate a change in performance, this study would have had 80% power to detect a ratio of 3:1 or greater in terms of the proportion of individuals with improved performance vs. worse performance.

## Results

Characteristics of participants are shown in Table 1. All subjects across all literacy levels correctly interpreted labels with instructions to take medication with water (label A), with food (label C), or not in conjunction with alcohol (label D), regardless of whether they were accompanied by visual illustrations. Participants' interpretations of label B and label E are shown in Table 2. A large number of responses were only partially correct. In the case of label B, participants often failed to note the intensifying effect of alcohol or the need to avoid operating a car or machinery. In the case of label E, participants often failed to note the recommended hours of separation between meals and medication use.

**Table 1:** Characteristics of Study Participants (N = 130)

**Characteristic**	**Number (%)**
Sex	
Male	57 (44)
Female	73 (56)
Age Group	
Under 25 years	25 (19)
25 to 39 years	40 (31)
40 to 64 years	51 (39)
65 years and over	14 (11)
Native Language	
English	92 (71)
Other language	38 (29)
Highest Educational Attainment	
Less than high school	5 (4)
Some high school	8 (6)
Completed high school	35 (27)
Post-secondary	82 (63)
REALM Score	
Grade 0 to 6	6 (5)
Grade 7 to 8	29 (22)
Grade 9 and above	95 (73)

**Table 2:** Label interpretations without and with illustrations.

	**Without Illustration**	**With Illustration**	P-value*
	Number (%)	Number (%)	
Interpretation of Label B			
Incorrect	23 (18)	29 (22)	
Partially Correct	63 (49)	57 (44)	
Completely Correct	44 (34)	44 (34)	
Change in Interpretation of Label B			
Improved	...	6 (5)	
No Change	...	113 (87)	0.33
Worse	...	11 (9)	
			
Interpretation of Label E			
Incorrect	13 (10)	14 (11)	
Partially Correct	46 (35)	44 (34)	
Completely Correct	71 (55)	72 (55)	
Change in Interpretation of Label E			
Improved	...	9 (7)	
No Change	...	112 (86)	1.00
Worse	...	9 (7)	

* Using the sign test to assess whether there was significant improvement or worsening in the interpretation of the label with the addition of the illustration.

The addition of visual illustrations in labels B and E resulted in some changes in interpretations (Table 2). With each label, however, the number of subjects whose performance worsened was approximately equal to the number of those whose performance improved. Thus, for both labels B and E, the addition of illustrations did not significantly improve or worsen the overall accuracy of label interpretation (Table 2). Most subjects whose performance worsened did so because they focused exclusively on the meaning of the illustration (for example, "drowsiness" or "empty stomach") and neglected the accompanying written information. This resulted in partially correct answers, as described above. Most subjects whose performance improved appeared to have attended to the written information on the label.

Sex, age group, being a native English speaker, and health literacy level were not significant predictors of improved or worsened comprehension when illustrations were added. It is important to note, however, that this study was not powered to detect such differences among subgroups of participants.

## Discussion

We found that the illustrations selected for examination in this study provided little or no benefit in improving patients' comprehension of labels informing them how medications should be taken or their potential side effects. Participants in this study had perfect comprehension of three relatively simple labels when they were presented without visual illustrations, leaving no opportunity for the addition of illustrations to have any positive effect. In the case of two more complex labels that a number of patients had difficulty fully understanding, the addition of illustrations had positive and negative effects on comprehension in approximately equal numbers of patients.

Our finding of mixed effects of illustrations may be explained by a number of factors. The illustrations on the labels we presented may have been ambiguous and failed to clearly convey a specific message, or at worst they may have been misleading. For example, the illustration showing the outline of a stomach could not be understood without some knowledge of human anatomy. Even if it were understood, such an illustration conveyed only one component of a more complex message. Furthermore, the illustrations may have captured the patient's attention, distracting him or her from the full message contained in the accompanying writing. This phenomenon might occur regardless of a patient's level of health literacy.

This study has several limitations. We only examined patients in urban family practice units associated with an academic teaching hospital. Because the vast majority of our study sample had an adequate or high literacy level, our data do not support any conclusions regarding the effect of medication label illustrations in patients with low literacy. In addition, our relatively small sample size and the large proportion of subjects whose performance was unchanged with the addition of illustrations raises the possibility of type 2 error, that is, a failure to detect true differences due to inadequate power. We used a selected set of medication labels to provide a range of complexity in terms of both text and illustrations; because our choice of labels was non-random, our results may not accurately represent all of the illustrations in use or their effect on comprehension. The size and readability of the images we used may have affected our results. The use of enlarged versions of the labels may have enhanced their readability, and participants did not undergo formal screening for visual acuity. Some degree of sequencing bias may have occurred, since the labels were presented in the same order for all subjects. Finally, we did not obtain qualitative data through detailed interviews that might have allowed us to clarify how participants perceived the meaning of the illustrations and how the participants related the illustrations to the text.

## Conclusion

Pictorial illustrations can improve comprehension of medication labels if the illustrations and text are well-matched to each other and are appropriate to the educational and cultural background of the user [4,7]. However, two older studies found that the use of symbols did not improve understanding of prescription instructions compared to written directions alone [5] and that comprehension of prescription labels was unaffected by the use of auxiliary labels (different from those used in this study) [10]. The inconsistent results of previous research in this area are not entirely surprising, given significant variation in the quality of illustrations and their ability to act as a complement to written instructions to improve comprehension.

Thus, while illustrations can be effective in improving patients' understanding of how their medications should be taken or their potential side effects, physicians should not assume that this is always the case. At least some of the images currently in use on prescription medication labels do not appear to be helpful, and the effectiveness of such illustrations should be further evaluated. Given their potential to confuse or distract patients, an alternative would be to replace illustrations with a common symbol, such as a large exclamation mark, to reinforce the importance of the accompanying text. Additional research is needed to better understand the cognitive processes that affect comprehension when written and pictorial pharmaceutical instructions are combined, and to develop more effective communication strategies to improve patients' understanding of the proper use of their medications [11].

## Competing interests

The author(s) declare that they have no competing interests.

## Authors' contributions

SWH participated in the design of the study, data analysis, and drafting of the manuscript. CQNT participated in the design of the study, data collection, data analysis, and drafting of the manuscript. NK participated in the design of the study, data collection, data analysis, and revision of the manuscript.

## Pre-publication history

The pre-publication history for this paper can be accessed here:


